# Practical Observations on the Convulsions of Infants

**Published:** 1826-07

**Authors:** 


*Practical Observations on the Convulsions of Infants.* By JOHN NORTH, Surgeon-Accoucheur, M.R.C.S.L. &c. &c. &c. 8vo. pp. 294, London, 1826.

AFTER adverting, in a concise preface, to certain exclusive views regarding the pathology and treatment of juvenile convulsions, our author states as a general principle, and we have long admitted the same doctrine, that these affections are rarely if ever *idiopathic*, in the strict acceptation of the term: they are *symptomatic* of most of the diseases of infancy, and shall consequently be frequently prevented by close attention to the first deviations from health, and the timely application of treatment which must vary with the constitution of the child, and the particular derangement or disease of which convulsion is a symptom, or on which it is likely to supervene. He considers the phenomena exhibited during the paroxysms, as claiming less of our attention than the insidious derangements of health which precede the occurrence of the seizures, or the consequences that result from their frequent repetition: his remedies, of course, are little different from what should be employed in many and various modifications of infantile disease. With these remarks, we enter on his volume: it consists of six chapters, each of which, in its order, we shall bring under our readers' consideration.

CHAP. I.—*On the Frequency, the Causes, and Symptoms of Infantile Convulsions:—the Prognosis, and Appearances observed on Dissection.* Infants are particularly subject to convulsive affections in every region of the habitable globe. In confirmation of this fact, Mr. North adduces the return made by Dr. Clarke in 1793, in which it is stated, that of 17,650 children, born in the Lying-in Hospital of Dublin, a sixth part died during the first year of their existence, and that 19 out of 20 fell victims to convulsions. Dr. Lange has also recorded, that at Copenhagen, during a period of 13 years, no less than 12,769 children perished from epilepsy: this physician attributed the prevalence of the disease to the luxurious and effeminate education practised in every class of society. Our author has not advanced any thing new or definite in illustration of this important question, but holds it to be a doubtless truth, that the death of a child is occasionally ascribed to the accidental occurrence of a convulsive paroxysm, when it was destroyed by some other disease, of which that was comparatively an unimportant symptom. He also regards it as being undeniable, that a vast number of children are suddenly cut off by an unexpected attack of convulsions, which not unfrequently occurs when no previous derangement of health could be discovered, or at a time when the malady, under which the child was labouring, began to assume a less severe aspect, and to justify a confident and favourable prediction of the result. From p. 4 and 5, we take an extract which contains two statements, at least, that merit attention.

"Although," he says, "the convulsions of children are *in most cases symptomatic* of some other disease, they are of sufficient importance to admit of a separate consideration, inasmuch as they not unfrequently destroy life in a few moments, when it was not endangered by the malady which excited them. Notwithstanding, however, the universal admission of the fact, that convulsions are always to be regarded with apprehension, I am inclined to believe, from frequent personal observation, that no cases are dismissed in general practice with less attention than those which pass under the convenient term of Convulsions. It is, indeed, a source of frequent lamentation with physicians, and not unfrequently of neglect,—which is so commonly the offspring of despair,—that the diseases of children are particularly difficult to detect, from their inability of describing their sufferings. I consider this difficulty more imaginary than real. He who will take the trouble to regard with attention *the looks, the gestures, and the general demeanour of children in health, will have but little difficulty to detect the first advances of disease;* although considerable experience may be required to determine the precise nature of the malady, or its appropriate treatment." 5.

Practitioners ought at all times to impress on the minds of parents the necessity of attending to the slightest convulsive movement in young children. With reference to this, Mr. N. observes, that attacks of this kind so commonly take place that they are not unfrequently looked upon with indifference, until their frequent repetition has strengthened the natural or acquired predisposition to their occurrence. The general axiom, he says, of "*remove the cause and the effect will cease*," is inapplicable to many derangements of the animal economy, and more particularly to those of the nervous system. When convulsions have once taken place, even in the most healthy subject, the brain and nervous system retain for a long and indefinite period such a morbid sensibility, that a return of them is to be apprehended from the most trifling causes, long after the *exciting cause* had been entirely removed. Nevertheless, he adds, what is precisely the change which is effected upon the brain and nervous system, or in what consists the difference between a brain thus predisposed and one that has never been subjected to a shock capable of producing disease, has never yet been satisfactorily explained, and probably never will be so: many children have died with all the symptoms of hydrocephalus, accompanied by violent and repeated paroxysms of convulsions, and, after death, no trace of disease could be detected in the brain and its membranes. Passing from these observations, Mr. North proceeds at p. 9, to enumerate such of the anatomical and physiological peculiarities of the animal economy during the infant state, as tend to explain the origin of the aptitude of infants to convulsive affections. We quote the following.

"Until the frail mind," he says, "and body of the infant are accustomed by habit to have their powers acted upon with impunity, the most hazardous susceptibility must necessarily exist. The muscles during infancy are pale, soft, and fragile; their contractions are quick, frequent, and feeble; and the external surface of the body is endowed with a very high degree of sensibility, in consequence of the nerves being covered only with a very fine thin cuticle. Hence, from very slight impressions arise very powerful effects. The circulation of the blood is very rapid, the arterial pulsations nearly double those of the adult. The capillary circulation is also infinitely more active. The lymphatic system exerts a more powerful influence upon the general economy of the infant than upon the adult. The muscular fibre as well as the skin is highly sensitive. The nerves are large in proportion to the size of the body. They resemble medullary pulps. Both the cerebral and ganglionic nerves are much more strongly developed in relation to the body than at any other period of life. The brain is large, and the nerves which proceed from it of a very considerable size. As we advance in years the coats of the nerves and the muscular fibres become firmer, and our susceptibility to external impressions is consequently diminished. Hence it is, than in proportion as we advance in years convulsions are less likely to take place. They sometimes occur during the period of youth. In the adult they are rare; and they scarcely ever happen in old age. The sensations of a child are quick but transient. When any reaction takes place in the system, it is powerful and sudden, and coincides with the general mobility:—motion, indeed, is the language of an infant." 11.

Many writers affirm, that children born with large heads, or whose heads increase in size disproportionately to their bodies, will have convulsions: Mr. N. has seen such seizures occur very frequently in children with small heads: this, however, does not bear directly on the former proposition: we ourselves have known hundreds of infants with large heads, in whom even the trace of a tendency to convulsions, was never discerned. It may be predicted with certainty, that the child born with a large head shall, if his brain continues healthy, be a powerful man; but we do not know one reason by which we should be induced to regard this state as indicative of a predisposition to convulsive affections. Mr. North declares, that the children of parents, who marry at too early or too advanced an age, are more susceptible of convulsions than the progeny of those persons who marry in the prime of life. This we conceive to be a very important fact in the natural history of man; and, of course, must be a deduction from extensive and greatly diversified observation: a sketch, however brief, of the manner of conducting such an inquiry would have been acceptable. No reason exists, in the judgment of our author, why a disposition to convulsive diseases should not be transmitted from parents to their offspring, as well as a disposition to other maladies. The children of women in high life, he says, who enfeeble their health by late hours, hot and crowded rooms, and irregular diet, are *undoubtedly* more disposed to convulsive affections than the children of those females who are regular in their mode of living, and who enjoy the calm tranquillity of a country life. This last dogma has been repeated, for ages, and we fear without consideration: indeed we do entertain very strong doubts of its accuracy, and would appeal to the experience of "rural" practitioners for a determination of the question. Here we may transcribe Mr. N's definition of a convulsion.

"When," he says, p. 15, "there is either an alternate and involuntary contraction and relaxation of the muscles, or a permanent contraction of the muscles, convulsion exists. The contraction may be so trifling as scarcely to attract attention; or it may be exerted in the highest possible degree which the muscular structure is capable of supporting without rupture of its fibres. When the muscular contraction is permanent, it is then termed a *tonic convulsion*. In this species, the involuntary contraction of the muscles renders them motionless, and incapable of renewing their motion until the entire cessation of the convulsion. When there is an alternate contraction and relaxation,—a rapid succession of irregular action and rest in the muscles affected,—the term *clonic convulsion* is employed."

In remarking on the nature of convulsions, Mr. N. observes, that the seat of the disease is most commonly the eyes, the features of the face, the upper and lower extremities, and the respiratory muscles. Although the preternatural movements of the face may sometimes be exceedingly slight, it perhaps never happens, he thinks, that the features retain their natural tranquillity of expression, while other parts of the body are convulsed. It is stated at p. 19, that neither fever nor *disturbance of the intellectual functions* forms a part of the symptoms of a paroxysm of simple convulsions: a child may not be able to hear during the paroxysm; but this, it is said, is not a proof that its faculties are destroyed. Great circumspection, we think, ought to be exercised in uttering doctrines founded on the manifestations of intellect in children; for, in very many instances, we have not the means of judging of the integrity of the intellectual functions during the first stages of life. According to our views, which we state respectfully, there is always a *disturbance*, both of the sentient and intellectual functions during the convulsive paroxysm, and the *degree* of this disturbance is *generally* commensurate with the *intensity* of the seizure: often, indeed, there is an interruption, a true suspension of these functions, without a pledge or security that they shall ever be resumed.

Mr. N. owns much difficulty in establishing clear distinctions between the various kinds of nervous affections: in many cases, he says, they pass into each other imperceptibly, and the line of distinction is scarcely to be defined. He recognises a strict analogy between epilepsy and simple convulsions, and declares the muscular system to be, in each disorder, affected in a very similar manner. We were not prepared to meet with this declaration, and confess our inability to reconcile it with the facts of general experience, and with Mr. N's own statement, that "neither fever nor disturbance of the intellectual functions forms a part of the symptoms of a paroxysm of simple convulsions."

With two hints on our ignorance respecting the periodical return of the accessions of many nervous diseases, on the violence of convulsive action, on subsultory startings, and on palpitation, Mr. North goes on to consider the "proximate cause or nature" of convulsions, concerning which, he acknowledges that we are, and probably shall ever remain, in doubt. We pass the first part of his observations on this head, with a recommendation of them to the reader's attention, and quote the following.

"It is undoubtedly," he observes, p. 32, "too much the custom of the modern system of education to stimulate the infant intellect to premature, and therefore, prejudicial exertion. The recommendations enforced by Struve should never be forgotten; and if they are forgotten by parents, it is the imperative duty of the medical practitioner to point out the necessity of complying with them. We should operate upon the tender intellect of a child by the gentlest progression. It must surely be much more judicious to complete the instrument previous to its use, than to employ it in an imperfect state. It is the same with children as adults. In the cultivation of the mental powers we are always to bear in mind the capability of the individual to answer the demands which are made upon him for exertion. It is not only irrational, but it is frequently destructive, to impose either upon the mind or body, but particularly upon the former, a load which it is incapable of supporting. It may be a source of consolation to those parents who are too apt to lament any apparent loss of time in the very early periods of life, to remember that early acquirements are not to be gained without destruction of health, and that the future progress and mental powers of the individual depend upon the foundation which is laid in infancy by judiciously adapting the studies of the child to its age and constitution. By premature efforts to improve the powers of the intellect, the organ in which they reside is exhausted. The practitioner, then, cannot too forcibly reprobate the pernicious enforcement of precocious studies. The injurious effects arising from the folly and false vanity of parents, who are ambitious of holding forth their children as specimens of extraordinary talent, are constantly presenting themselves to our view in a train of nervous symptoms, and of susceptibility to ordinary impressions, which frequently pave the way to decided paroxysms of convulsions."

Experience supports our author in allowing, at p. 41, that the brain is directly or indirectly irritated in most cases of convulsions: nevertheless, although this irritation may last but for a moment, and be so trifling as to produce no serious derangement of the cerebral functions, much less any alteration of the cerebral structure, yet still, he adds, it may be kept up by a repetition of the exciting cause, and produce irremediable mischief. At the same time, he cannot admit irritation of the brain to be the proximate cause of all convulsive affections; for, in some cases of local injury, they must arise independent of the "mediation" of the brain: neither can he conceive how the convulsive affections which arise in plethoric habits, and require abstraction of blood, and those that occur from the accidental loss of blood,—for it is an established fact, that every animal which dies from hæmorrhage suffers violent convulsions,—can alike proceed from cerebral irritation. He admits, however, that in most cases of the kind, there is some *functional* disturbance of the brain, and that *organic* disease of the brain may originally exist as their cause, or be consequential to them; but, he adds, we must pause before we assent to, or shape our practice by, the unlimited statement, "that, in every case of convulsion, the brain is at the same time *organically* affected, either directly or indirectly." In the subjoined extract, there are several observations leading to good and discriminate practice, and consequently most worthy of serious reflection.

"Both in children and adults," says Mr. North, p. 47, "the effects which arise from any given irritation will depend upon the particular constitution and temperament of the individual. In one, local pain unconnected with general disturbance may ensue; in a second, an attack of fever may arise; and in a third, convulsions with or without pain or febrile movement. With whatever train of symptoms an infantile disease commences, or in whatever part of the body derangement of function or disease of structure may primarily be situated, the irritation endured by that part may be reflected upon the brain, and convulsions may follow as a symptom of the cerebral reaction upon the muscular structure. The probability of the occurrence of convulsions will be determined by attention to the particular constitution of the patient. The character of the original disease may vanish, and the treatment at first required must be changed for one more directly appropriate to the transition which has taken place. But although we are to bear steadily in mind the possibility and even probability of convulsions being produced by some serious cerebral disease; it is equally important that we should not hastily determine that the brain is organically or even functionally affected, because a paroxysm of convulsions occurs, or prognosticate the speedy effusion of water in the ventricles, unless enormous doses of calomel are prescribed and repeated bleedings are had recourse to. Such a mode of practice has been recommended by high authority in several cases of convulsions which I have watched with much attention; and at the same time it has been confidently asserted, that hydrocephalus would ensue if any part of the plan of treatment were omitted. A much more moderate, and I think more rational plan has been adopted, and the children have perfectly recovered. Unless they arise from mechanical violence or intense moral impressions, idiopathic affections of the brain are, I conceive, very rare in children. I by no means agree with the opinion, that when convulsions are symptomatic of encephalic inflammations, it is almost always the case that some evident cause has been applied, which has acted mediately or immediately upon the head, and from which inflammation almost certainly arises. Convulsions quite as frequently occur during inflammation of the brain, which is produced by sympathy with some distant part that is highly irritated. It is also said by the same authority, that there is a well-marked difference in the convulsions themselves, when they arise from affections of the head. It appears to me, when a paroxysm of simple convulsions is once excited, whatever may have been the cause of it, that it is essentially the same, although it may differ much in its degree of violence and duration."

Mr. North condemns, as a source of fatal error, the practice of seizing upon some individual symptom, such as slight squinting, partial convulsions, or a crouping noise in breathing, as the ground for determining that water in the head will inevitably be the consequence, unless a particular and formidable treatment—large doses of calomel and profuse depletion, be employed: each and all these symptoms, he says, may arise when we have no reason to suspect any affection of the brain. Convulsions, he adds, in many instances are altogether local; they are confined to particular muscles, or particular sets of associate muscles, and have no effect whatever on the brain so as to disturb its energy. His observations on the convulsions of new-born infants and their causes; on the trismus nascentium; on those which proceed from inflammation of the cerebral membranes and spinal marrow; from tumours; depositions of bony matter and sanguineous effusions in the brain; from blows and wounds of the head; and from the desiccation of cutaneous discharges, attest the extent of his practical experience and acquaintance with medical history. At p. 62, he says, Nature appears frequently to labour under considerable difficulty in the production of various cutaneous affections; and, previously to the occurrence of eruptive diseases, paroxysms not unfrequently take place. In such cases, he adds, they are generally considered as a favourable omen; and, from his own experience, he should infer that the opinion is well founded, provided the eruption makes its appearance at the usual period from the commencement of the premonitory symptoms. Some things in the following extract, will probably be regarded as singular by many persons in the profession.

"That the brain," p. 63, "does sympathize strongly with affections of the skin is a fact which is so frequently forced upon our observation, that no laboured confirmation of it will be required. There are some circumstances, however, connected with this admitted truth, that may admit of a moment's observation. When inflammatory action takes place, it is confined to the capillary system, which is independent of the cerebral nerves; but the latter are excited by the relation which they hold with the former; and if this excitement reacts with force upon the brain, convulsions may be produced. The convulsive phenomena are more likely to occur at the time of the appearance of the eruption, because it is then that the greatest degree of irritation exists. We have observed that some respectable authorities look with suspicion upon the occurrence of convulsions previous to eruptive diseases. This discrepancy of opinion may perhaps be reconciled by reflecting, that whenever an irritation of the brain takes place in consequence of the sympathy of that organ with a distant part, that such sympathetic irritation, particularly in very irritable subjects, may pass into an independent irritation of the brain, which may be followed by very serious consequences, or even the death of the patient. Here then, the danger will be depending not upon the eruptive disease, which was the prime mover of all the disturbance, but upon the affection of the brain, which has been superadded. The opinion, therefore, that is so generally entertained, does require modification. Instead of asserting that convulsions which precede eruptive diseases are always harmless, we should only consider them so when they are produced by an excitement of the brain, which appears to be purely sympathetic."

Intestinal worms are generally allowed to be a frequent cause of simple convulsions or epilepsy in children: our author is not inclined to deny, that convulsions may sometimes arise from this source; but he believes much less frequently than is usually imagined. He has seen many "worm cases," but does not remember a single instance where convulsions appeared to depend upon the presence of worms in the intestines, or to be relieved by their being discharged. Our experience has been different; we have treated many cases in which we were sure that the convulsions originated from excitement induced by these parasitic animals, and subsided on their being expelled from the bowels by appropriate, especially terebinthinate remedies: the statement of varied observation ultimately leads to the establishment of truth.

We need not follow Mr. North very closely through his enumeration of the symptoms which distinguish the convulsive affections. He holds it to be always useful, in diseases, to examine the position of the limbs during sleep, particularly the sleep of children. If they deviate from the ordinary degree of flexure to the more straight position, there is generally some irregularity "in the state of tone and, of course, in the vital influx." Upon viewing the position of a child during sleep whom, from the occurrence of these symptoms, we consider disposed to convulsions, we shall frequently find, he adds, the limbs rigidly extended, the great toes and thumbs being turned inwards. Stretching of the limbs, it is true, is both in adults and children a natural action, exercised to restore muscular equilibrium; but, he concludes, in connexion with several of the other premonitory symptoms, it must be considered as a strong indication of a tendency to convulsive movements. Speaking of hiccup as a premonitory sign of more general convulsions, he states that he has never seen this symptom alarmingly severe in itself in children, although it is sometimes troublesome from its frequency and long continuance. In them, he says, its frequency is doubtless to be attributed in many instances, to an excessive quantity of food stimulating a weakened stomach. At p. 101, we meet with these sentiments, and copy them.

"A plethoric state of constitution increases the natural aptitude of the child for convulsions. But we must guard against being influenced by an erroneous opinion which is very general. There are many practitioners, and some of no mean celebrity, who assume, that in every case of convulsion there is either a local or general increase of the actions of the arterial system, which demands for its relief the abstraction of blood. It is, therefore, the more necessary to insist upon the frequent occurrence of convulsions from debilitating causes, and from a state of nervous irritability, without any vascular excitement either local or general. It has been said by Haller, and the doctrine has been adopted and repeated by Bichât, 'that the vital force manifests itself in two opposite states;—in paralysis, and convulsions. The first is the sign of *diminished energy*; and the second, of *augmented energy*.' Such an assumption is, I apprehend, contrary to fact; and if indiscriminately acted upon, must be followed by injudicious practice. Paralysis frequently takes place in such a condition of the general system as to require depletion for its relief: and, although the latter part of the proposition is often true, it must be admitted that it has many exceptions, and that 'augmented energy' is not the necessary attendant of convulsions, unless indeed the term refers merely to the increase of muscular action. It is worthy of remark, that every animal which dies from loss of blood is attacked with violent convulsions during the last moments of its existence. This fact is daily exemplified in slaughter-houses. Puerperal women, who suffer considerable hemorrhage from the uterus, are almost invariably convulsed. There can surely be no 'augmented energy of the vital force' in these cases; for it must be observed, that convulsions occur before any reaction takes place in the system weakened by excessive bleeding." 103.

Into this long chapter Mr. North has introduced a multitude of sensible remarks, which are chiefly practical, and on that account more valuable: the most experienced of our readers may peruse them with advantage. Towards the conclusion he remarks; we have yet to learn how to determine positively when convulsions do arise, and when they do not, from organic lesion of the brain, or from effusion of water into its ventricles. He thinks we have little reason to apprehend danger when the convulsive attacks are slight and of short duration, and succeeded by the natural cheerfulness of the child. On the contrary, he adds, a repetition of the attack is to be dreaded when the paroxysms are of long continuance, gradually increase in severity and violence, and leave the child dull and heavy. In all cases he himself witnessed, where the infant was destroyed suddenly during convulsions, the dark colour of the face and neck, and the almost stertorous breathing indicated a state nearly allied to apoplexy in the adult. He failed of procuring a dissection of such subjects: we have made more than thirty, and invariably found a very loaded state of the sanguineous system in the head, with more or less effusion of lymph in the ventricles of the brain: in several we discovered considerable extravasation of blood from a ruptured vessel.

Chap. II.—*Treatment of Convulsions.*—Mr. North very judiciously opens this chapter by reminding the reader that, as the causes capable of producing convulsions in children are almost innumerable, so their treatment must be ever varying: it would, therefore, he says, be a useless effort to endeavour to lay down a determinate mode of practice for every modification of these convulsive affections: the general principles upon which our treatment must rest can only be expected. We shall not, on this occasion, undertake a systematic exhibition of his doctrines on this important branch of practice, but confine ourselves to a few selections and extracts, accompanied with incidental remarks. We prefer this, as the method best adapted to induce our readers to study the work itself with that care and attention, which the candour of its author, and the excellence of his precepts indisputably deserve.

According to Mr. North's experience, the severity of convulsions in children is frequently much increased by the irritation arising from repeated attempts to administer medicine internally during the paroxysm. He prefers opiate frictions upon the chest and abdomen, as being then more serviceable than any internal remedies. It can never, he thinks, be improper to inject a purgative glyster while the fit prevails; and, if there are evident marks of a tendency to sanguineous congestion in the head, he advises abstraction of blood, by opening the jugular vein or cupping the temples: to apply a few leeches, according to the common custom, is a loss of time, if bleeding be really required. Let practitioners ponder well the practical observations we now transcribe.

"It is not an uncommon error," says Mr. North, at p. 138," for practitioners to estimate the strength of the patient by the violence of the convulsive movements, and to determine the propriety and extent of bleeding by this deceptive criterion. It is to be remembered, however, that no convulsions are more violent than those which arise from hemorrhage or other debilitating causes. The most violent paroxysms of convulsions I ever witnessed occurred in the person of a weak and delicate woman, who had suffered from uterine hemorrhage after labour. We are not, therefore, to abstract blood during a convulsive paroxysm merely with the intention of relieving its violence. We must be guided by those appearances which have been already stated to indicate an undue and dangerous determination of blood towards the head: but if in every case we bleed during the convulsions either of adults or children, however strong may be the popular prejudice in favour of the practice, the life of the patient will frequently pay the forfeit of our unjustifiable compliance or our want of judgment. It must be evident that the tender constitution of a child cannot bear the long-continued and violent convulsive struggles which we frequently witness, without much subsequent exhaustion."

Willis and others have said, that if blood be taken during a paroxysm of convulsions, it coagulates instantly like butter: Mr. N. however, has never observed this phenomenon, and confesses his doubt of the fact altogether, but regards the speedy occurrence of concretion on the effusion of blood, as a reason sufficiently powerful for the discontinuance of depletory measures. Notwithstanding Dr. Currie asserted, from eight years' experience, that the cold bath is very efficacious in removing the convulsions of children, from whatever cause they may arise, that it stops the fit, and gives time for the application of other remedies; yet our experienced author declares, that he should be unwilling to plunge a child of a very delicate and enfeebled constitution into a cold bath, while labouring under a paroxysm of convulsions: it would appear to him, he says, to be a hazardous experiment. What we next extract, contains matter of momentous import:—

"If the child," says our author, p. 145, "is robust and of a plethoric constitution, with the head hot, and disproportionately large, the carotids throbbing, the countenance flushed, the eyes sparkling and projecting from the orbit; if he sinks into a state nearly approaching to coma, after any unusual agitation, we have reason to fear a hazardous determination of blood to the head, and must proceed accordingly. Under such circumstances, a state very nearly resembling that of apoplexy in adults may follow. The symptoms may be analogous between the two diseases; but the appearances on dissection will rarely be found the same. Baumes was of opinion, that every child who died during a paroxysm of convulsions, died from apoplexy. If the term Apoplexy, however, is to be limited to extravasation of blood in the brain, it is a very uncommon disease in children: but, if it is extended to every case of cerebral compression, it is of very frequent occurrence; for the same phenomena will often be the result of inflammation of the membranes of the brain;—of effusion of water into the ventricles, &c. Local or general bleeding, or perhaps both, may be required according to the intensity of the symptoms and the constitution of the patient. The mode in which we abstract blood is of considerable importance. I must be allowed to declare my utter want of confidence in the practice which is recommended by almost every authority. I allude to the application of a few leeches to the temples. I have never seen well-marked symptoms of determination of blood to the head in children removed by leeches, however freely they were applied. Their application never fails to annoy the little patient considerably, and their effect is not to be relied on. Blood should be taken from the jugular vein, or we should have recourse to cupping upon the temples, or behind the ears. Cupping is at once an elegant and efficient mode of abstracting blood, provided, indeed, the operation is adroitly performed, which it most assuredly will not be, unless it is entrusted to those who are constantly employed in that particular branch of surgery."

At the risk of enlarging our article with extracts, we must give the following, professing our entire approbation of the author's sentiments.

"The bowels," p. 152, "should be freely acted upon by proper doses of calomel combined with jalap. Laxative glysters are to be considered as useful auxiliaries. I entirely disapprove of the practice of administering in such cases large and repeated doses of calomel every two or three hours, from some hitherto undefined, and, I believe, imaginary notion, that this remedy is capable of acting as a specific in convulsive affections, independent of its purgative properties. I am confident that the constitutions of children are frequently ruined by the heedless and indiscriminate manner in which this powerful medicine is employed. The practice requires to be more strongly deprecated as it is not only pursued, but taught, by very high authority in this country. For what purpose, it may be asked, can calomel in two or three-grain doses every three or four hours be prescribed in a case of convulsions? I presume, the intention must be to stimulate the lymphatics, and to remove any fluid that may have been effused into the cerebral cavities. But the occurrence of convulsions, it is to be remembered, is no proof of any effusion of water, or of any disposition to such effusion in a great majority of cases."

At p. 156, Mr. North discusses the advantages to be expected from the proper and assiduous application of cold to the head, in cases where symptoms of determination of blood to the brain, are present. He directs the whole head to be completely wetted with a large sponge soaked in spring-water, which should be frequently changed; or to have pounded ice, in a bladder, applied over the same parts. When the child becomes pale and the head cool, these applications should be discontinued, and renewed when flushing of the cheeks and heat of the head, indicate a return of the vascular excitement: he inculcates, at the same time, a careful support of the natural heat in other parts of the body. Many of the French physicians put the little patient into a warm bath, while the ice is being applied to the head, and the plan, says Mr. N., I have no doubt is very judicious. In cases of this kind, we have for many years, directed the practice of immersing the child's lower extremities in a warm bath, while cold water is poured, in a gentle stream, on the head and cervical spine, by an assistant; and, during this process, the countenance is attentively watched, and the pulse in both wrists observed. When paleness and collapse of the face supervene, and the arterial actions decline or intermit, the effusion is suspended and the head and trunk covered with a dry cloth; but, so soon as the first signs of a re-action are detected, the process is resumed, even to the third or fourth time, till its good effects shall be decisive and manifest in the suppression of all convulsive motion.

Mr. North is not partial to the application of blisters to children, but apprehends that the principle of counter-irritation upon which they have been recommended to be applied to the lower extremities, has been unjustly ridiculed. In many instances, where there was evident determination of blood to the head, *without any general excitement*, he has obtained the best effects from blisters to the calves of the legs, or between the shoulders. Blisters to the head are decidedly prejudicial; for, he affirms, they certainly keep up a discharge from the integuments of the head, which can only be maintained by increasing the arterial action within the cranium. This, we suspect, is one of those little theories by which even great minds sometimes allow themselves to be amused. If, under such circumstances, there must be a *local* increase of arterial action, why should the nearest vessels escape the excitement? Externally to the cranium, there is a sufficiency of arteries surely, by whose increased activity, a discharge of the kind could easily be supported, while those of the brain should have their energies moderated or depressed. Might it not be expected, then, that there should be an abstraction of excitement from the cerebral vessels, proportionate to the degree of exaltation imparted to those in the integuments of the head? But all this, of course, is pure assumption; and, if Mr. N. has one fact in support of his conclusion, this must certainly be preferred to a thousand arguments however plausible these at first sight may appear. There is much good sense in our author's rules for the exhibition of opiates to children: we transfer them to these pages.

"I am aware," he observes, p. 165, "of the strong objection that exists to the employment of opiates in the diseases of children. To a great extent I admit that these objections are well founded; but I am convinced that the popular abuse of narcotic medicines, the mischievous effects of which are so frequently presented to our view, has led to their too general and exclusive prohibition among practitioners themselves. Every thing is now expected from bleeding and purging and calomel; and not unfrequently these remedies are pushed to a destructive extent, when much more benefit would be gained by the tranquillizing effects of sedatives. We are not to deprive ourselves of a powerful weapon, be[illegible]e in the hands of the unskilful it may have proved the means of destruction rather than of defence. To manage the use of opium or other medicines of the same class adroitly, either in adults or children, when it is our object to subdue nervous irritability, is by no means an easy task. The effects of these remedies depend greatly upon the state of the constitution when they are exhibited, and upon the dose in which they are given. No abstract rules can determine the precise dose of a medicine; but with respect to the state of the constitution, it may perhaps be assumed as a general axiom,—that if there is any local disturbance or disease upon which the general irritability and restlessness of the little patient depend, we should endeavour to remove the local affection by proper means, before we venture to exhibit sedatives. In the administration of sedatives to children, we should always *commence* with the utmost caution, and progressively increase the dose. So variable are the effects of this class of medicines, particularly in children, that in each case we can determine only from the operation of the remedy the propriety of its continuance, or the dose in which it is to be exhibited."

At p. 202, Mr. North reverts to the subject of blistering children, with a great deal of good feeling, and sound philosophy. If convulsions occur during fever, he says, we are advised by many authorities, to apply blisters freely: he confesses, however, his ignorance of the principle upon which this practice is recommended. In many instances he has seen considerable distress, and aggravation of symptoms arise from blistering children in such circumstances; and, twice, he knew infants destroyed by the sloughing of ulcers, which could not be arrested. By repeated observation he has been led to adopt the opinion, that if blisters were *never* applied to children *in any case whatever*, much less evil would arise from the want of them, than is in common practice daily, or perhaps hourly, inflicted by this popular and painful application; except in the comatose state, he believes it rarely acts beneficially as an excitant. For squinting, he advises the child's being obliged to wear, for two or three hours every day, an ivory instrument over each eye, with a very minute aperture in the centre: the voluntary effort to look through the holes, without which, vision cannot be enjoyed, will occasionally restore the natural action of the muscles, and remove the deformity. He gives other methods, and lastly, that of Dr. Jurin, which is this: Place the child before you, and let him close the undisturbed eye, and look at you with the other. When you find the axis of this eye fixed directly upon you, bid him endeavour to keep it in that situation and open his other eye. You will now immediately see the distorted eye turn away from you towards his nose, and the axis of the other will be pointed at you: but, with patience and repeated trials, he will by degrees be able to keep his distorted eye fixed upon you, at least for some little time, after the other is opened; and when you have brought him to continue the axis of both eyes fixed upon you, as you stand directly before him, change his posture; put him first to one side of you, and then to the other. When in these different situations he can perfectly and readily turn the axis of both eyes towards you, the cure is effected.

Mr. N. at p. 216, says it may be worthy of observation, that M. Husson, a French writer, in his researches upon the vaccine disease, has recorded two cases in which the appearance of the vaccine vesicles entirely freed the patients from convulsive paroxysms to which they had been subject for several months, and which had resisted all the ordinary methods of treatment. For our own parts, we hold any communication from M. Husson in very low estimation indeed: he who could fill many pages of an 8vo. volume, to prove, by fiction and falsehood, that Dr. Jenner pilfered the first idea of vaccination from a Frenchman, is too contemptible to be censured.

Chap. III.—*On Infantile Epilepsy.* Epilepsy in children, our author thinks, is an extremely common disease: it is sometimes hereditary, but much more frequently acquired after birth. During the first four or five years of life, both sexes are equally subject to it, but, he says, after the age of seven or eight, it is certainly more common in females. Every cause, capable of producing a slight and even partial convulsive affection, we are informed, p. 220, may give rise, under particular circumstances of predisposition, to a well-marked paroxysm of epilepsy. From what he himself has observed, he should doubt whether epilepsy and simple convulsions be reciprocally convertible into each other: he never saw a case of epilepsy lose its distinctive characters and pass into simple convulsions. Dr. Parry, after Hippocrates, taught that epilepsy depends on excessive impetus of blood in the vessels of the brain, whatever may be its primary causes; but, in Mr. North's opinion, we have often no evidence of any such increased action in the cerebral vessels, even where the epileptic paroxysms have been frequent and severe. He also regards the treatment that is generally considered capable of reducing "excessive impetus," either local or general, as being such as would in many cases, rather aggravate the severity and increase the frequency of the attacks. Repeated dissections of epileptic patients have discovered lesions of the brain, to which the disease has naturally been attributed; but these appearances have not unfrequently also been detected both in children and adults who never suffered from epileptic or other convulsive maladies: besides, as Mr. North very properly asks, if the epilepsy had been dependent upon a cause which was constantly present, how can we account for its paroxysmal form?

Epilepsy is usually divided into the idiopathic and symptomatic kinds: in the former, its cause is presumed to reside in the brain, or the brain is immediately acted upon: in the latter, a part distant from the brain is originally irritated; it is infinitely the most common. In children, perhaps, the most fertile source of symptomatic epilepsy may be traced to derangement of the stomach and bowels: from this, at first, slight attacks of simple convulsions may succeed, which ultimately degenerate into epilepsy; or, an epileptic paroxysm at once occurs, without any previous convulsive malady of a milder nature. This is not surprising, says Mr. North, when we consider that the stomach is so abundantly supplied with nerves derived from the eighth pair and intercostal nerve, both of which have so striking an influence on the animal economy. In every case of epilepsy, he adds, the brain is affected either primarily or secondarily. There is as much variety in the duration as in the severity of an epileptic paroxysm: sometimes, it lasts only a few minutes; sometimes for several hours. The interval between the paroxysms is also very uncertain; and the danger is in proportion to the frequency of the seizures: when death ensues, it usually takes place in the state of coma and exhaustion both of body and mind, which successive attacks of the disease almost invariably produce. An epileptic paroxysm may occur several times in the day; and, although one patient may suffer repeated attacks without permanent injury, another may be quickly destroyed during the fit, or reduced to a state of idiotism: the severity of the paroxysm is generally more dependent upon the irritability of the child than upon the nature of the exciting cause. Medical history relates the cases of patients who were invariably seized with epilepsy from the influence of certain odours, from particular noises, or from the sight of certain colours: one child had always a paroxysm on observing any red object: the cases arising from the sudden sensation of fear, or from the sight of a patient in a fit, are most difficult of cure.—These sketches, from our author, are indicative of experience and research.

Mr. North's description of the premonitory and diagnostic symptoms is concise and graphic: his picture of a *strongly marked* epileptic paroxysm is elaborate and faithful; we give it insertion.

"The eyes," he begins, p. 230, "appear to project unnaturally, and are fixed; the eye-lids tremble; the ball of the eye is thrown upwards, so that only the conjunctiva can be seen; the face is swelled, becomes red, livid, or black, and is sometimes apparently in a state of ecchymosis: the features are horribly distorted by the powerful and irregular action of the muscles of the face: the lower jaw is sometimes firmly closed; at others it is forcibly separated from the upper jaw, and luxation of it is to be feared: the tongue is frequently thrust from the mouth, from which is discharged a frothy saliva; and if proper precautions are not adopted, the tongue may be severely injured when thus protruded, from the alternate contraction and relaxation of the lower jaw: the blood-vessels of the head and neck are excessively turgid: the head is thrown about in various directions, and sometimes becomes suddenly fixed: the whole body is agitated by the most violent convulsions, which may subside for a moment or two, to be again renewed with undiminished force. The same remission of the convulsive movements is also frequent during the paroxysms of simple convulsions. During this cessation, however, the expression of the countenance, and the gestures of even a very young child, lead to the conviction that it is sensible of its state of suffering. Not so in the abatement of the violence of a true epileptic paroxysm. The child either lies motionless and totally insensible, or rolls its eyes about with a wandering and unfixed gaze, without being attracted by the look of the mother or the nurse, and without the appearance of any degree of consciousness. The limbs of one side are commonly more affected than those on the other. In most cases, the child, when attacked, staggers and falls instantly to the ground. Occasionally, however, he remains fixed in the position in which he happened to be at the moment of attack: the head is moved from side to side with great rapidity, all consciousness being destroyed. An attack of epilepsy is sometimes marked by a sudden barking noise, arising from a convulsive movement of the pharynx. In hysterical women this symptom is very common, and, from the similarity of the convulsive paroxysms in Hydrophobia and Hysteria, may have given rise to the popular supposition of hydrophobic patients barking like a dog. Sometimes there is a trembling of the whole body, without well-marked convulsions of any part, which is followed by rigidity of all the frame, and total privation of sense. I have myself never seen this form of attack in infants. It is mentioned, however, by most authorities. The respiration is generally laborious. The breathing is sometimes stertorous; and the attack is then closely allied to, if it is not identically the same with, apoplexy in the adult. Symptoms of internal disturbance are frequently joined to the external and evident convulsions. The child vomits: the stools and urine are passed involuntarily. In fact, the diaphragm, stomach, bladder, and intestines, appear to be under the dominion of the same irregular and involuntary contractions as the external parts. After the termination of a paroxysm of epilepsy the patient is generally bathed in sweat. De Haen observes, that the perspiration has sometimes a very fetid smell, and is so abundant as to wet through the bed-clothes." 233.

CHAP. IV.—*Treatment of Epilepsy.* From the endless crowd of causes upon which epilepsy depends, it is evident that the indications of its cure must also be various and generally uncertain: many cases of the disease, indeed, in the present state of our knowledge, are totally irremediable. Impressed with this melancholy truth, Mr. North sets out, in this chapter, to point out the best measures for alleviating the severity of the disease, and for procuring as long an interval as possible between the paroxysms. He has stated these with unusual conciseness: they are very pertinent, and founded on principles exceedingly philosophical: we must, therefore, give them a place in the Journal.

"Whatever," he says, p. 240, "is capable of increasing the action of the heart and arteries, or of causing a determination of blood towards the head, should be carefully avoided. Violent moral impressions frequently induce a sudden and severe paroxysm of epilepsy, even in children who had previously exhibited no manifest disposition to convulsive affections. Where convulsions or epilepsy have once occurred, such mental emotions should be guarded against with additional care. The same observations that have already been applied to impress the importance of paying strict attention to the diet of children particularly predisposed to simple convulsions, refer with equal force to epileptic patients. The same principles must also guide our practice in the selection and continuance of purgative medicines, and in our general mode of treatment; although, it must be confessed, we are much less likely to succeed in our efforts to relieve epilepsy, however judiciously they may be directed. With every suitable precaution, however, both with respect to diet and the general management of the child, a determination of blood towards the head will frequently require to be repressed by the abstraction of blood. I should prefer cupping from the temples or behind the mastoid process. The jugular vein may generally be opened with facility and advantage. The quantity of blood drawn can only be determined by the effect produced. The head should be washed with cold water every morning, and on no account should the hair be allowed to grow either thick or long. Such is, in brief, the only treatment we can pursue, —the only precautions we can adopt in those cases of long standing and confirmed epilepsy, which we have reason to believe arise from causes over which we have no power, and which are accompanied by symptoms of determination of blood to the head. I have not spoken of the application of moxa to the spine, of blisters to various parts of the body, and of some other painful remedies, the use of which has been suggested by various authorities to prevent the return of epilepsy in children. In many instances of epileptic children I have known these experiments tried (in my opinion very unjustifiably) without any advantage. To such an extent has the rashness of some writers proceeded, that they have advised us to trepan the cranium, in the hopes of finding hydatids in the brain. The actual cautery has also been applied to the head. It is an easy, although not a harmless, amusement, to speculate in the closet, and to devise means for the removal of a disease, the origin and progress of which we picture to our minds according to the capricious influence of the moment." 243.

Mr. North next goes on to notice the value of different external applications, anti-epileptic specifics, and internal remedies; and, on this head, expresses himself with great candour and discrimination. He states, with De Haen, the necessity of paying strict attention to the symptoms which precede the paroxysm; for, he says, if we succeed in cutting them short, the fit is in most cases procrastinated. In confirmed cases of epilepsy, we are undoubtedly justified in trying any remedy that inflicts no suffering; but, Mr. N. declares, with much humanity as well as propriety, that he should not be inclined himself to add the torment of painful remedies to the severity of the disease, without more reasonable expectations of success than experience appears to promise. He has tried many of the internal remedies that have been proposed, as well as the external counter-irritants, but with little benefit. Oxyde of zinc, given for a considerable time, appeared to render the paroxysms less frequent: if the child is of a sufficient age to express its feelings, and can describe the peculiar sensations which indicate the approach of a paroxysm, he should, in most cases, exhibit an emetic of the sulphate of zinc: this, he adds, would not prevent abstraction of blood, if indicated. He has often seen both epileptic adults and children tormented for weeks and months by setons, blisters, tartar-emetic ointment and issues, but in no one instance has he known these things do any good. Judging from the results of his own observation, he concludes that counter-irritants have been extolled from speculative notions of the benefits they ought to produce; rather than from any practical proofs of their actual effects. He has never administered musk, assafœtida, castor or valerian, and thinks that, in infantile epilepsy, opium can rarely be admissible. When the disease is caused by excessive weakness, arising either from loss of blood or defective nourishment, he advises our proceeding very gradually in the application of invigorating remedies: when the attacks have not been very frequent, sometimes a permanent cure may be obtained by the carbonate of iron, sulphate of quinine, and the daily use of a bath, progressively reduced from the tepid to the cold temperature. Mr. N. deprecates all attempts at removing convulsive affections by exciting alarm; but, when they proceed from irritation, he would try the effects of the fear of punishment. When the epilepsy of infants is symptomatic of disease affecting the whole system, or depends upon some local irritation, as painful dentition, the remedies must, of course, be directed to a removal of the cause: inunction of the brimstone ointment upon the abdomen has been very warmly recommended by the German physicians, for the cure of the infantile epilepsy.

Chap. V.—*On a Spasmodic Affection of the Chest and Larynx in Young Children, accompanied by general or partial Convulsions.* Mr. North has treated many cases of this very distressing variety of convulsive affections; and, by this means, has been enabled to give an exquisite description of the phenomena by which it is characterized. We should fail of our duty were we to pass it with a cursory sketch: it is this;—

"The premonitory symptoms," says he, p. 254, "occur at an uncertain period; generally between the third and seventh month. At first they may not be sufficiently striking to attract the particular attention of the friends, although the practitioner, who has met with similar cases, may with much confidence predict from them the series of symptoms which is subsequently to be developed. When the child wakes from its sleep, the breathing is for some moments unusually accelerated, and is accompanied by that kind of noise which an increased secretion of mucus in the air-passages would produce. If the little patient has previously enjoyed a good state of health, the characteristic rotundity of feature observable in infants quickly undergoes a remarkable change; the countenance becomes anxious; the sides of the nose are drawn in; the face is pallid and emaciated; the child frowns almost constantly: when put to the breast, it sucks greedily for a moment, but suddenly ceases to do so, throwing back the head with violence. Whatever may have been the previous condition of the bowels, they now become constipated. A considerable time may elapse before any remarkable change takes place in these symptoms. A convulsive affection of the hand is usually the next morbid sign which excites attention. The child's thumb will be found constantly and firmly pressed upon the palm of the hand: the wrist and ancle-joints are bent rigidly inwards! the head is almost constantly thrown backwards, by which the anterior muscles of the neck are kept painfully upon the stretch. The inconvenience at the moment of waking is not now a mere acceleration of the breathing,—this symptom still continues in an aggravated degree,—but the noise accompanying the respiration has gradually assumed a very different character from that which at first marked it. Each inspiration is now attended by a loud crouping noise, which may be heard in an adjoining apartment: the chest and larynx appear to be painfully constricted; the heart palpitates violently; the child sobs, but never cries in its natural manner, during these paroxysms of suffering. So great is the difficulty of breathing, that it sometimes appears to be almost totally suspended for a few seconds. The countenance is then, usually, pale as in a state of syncope. Sometimes, but more rarely, it is dark, and the vessels of the face turgid as in apoplexy. The child has frequent attacks of convulsions, during which the features are much distorted. The whole body is sometimes, but more rarely, implicated in the convulsive movements The paroxysms vary in duration and violence. In a child, in whom the convulsions were very frequent and severe, the state of opisthotonos was so complete, that for many days the head and heels were the only parts which touched the bed. If with difficulty this apparently painful position was altered by the mother, it was quickly resumed. The anxiety of the countenance, which was at first only occasional, becomes in the progress of the complaint constant and very strongly marked. The brow is constantly knit. In the majority of cases no sustained febrile action is to be detected, nor is there usually any indication of particular determination of blood towards the head. I have lately, however, seen two cases in which were superadded to the above train of symptoms considerable febrile disturbance and much cerebral derangement, with evident determination of blood to the brain. In several instances I have known the firm contraction of the thumb, the rigidly bent position of the hand and foot, and the crouping noise in respiration, continue for many weeks without intermission. The child sometimes appears lively for a short period, and the countenance may be animated by a momentary gleam of cheerfulness; but it almost invariably awakens from its slumbers, however tranquil they may sometimes appear, with a convulsive paroxysm similar to that above described." 259.

Our author believes that this affection is, in many instances, immediately connected with painful dentition, and has procured instant relief by freely dividing the gums: the symptoms gradually subside on the appearance of teeth. In connexion with the other signs, there may be distinct evidence of cerebral derangement, and a necessity for prompt and vigorous treatment; but, in a great majority of cases, he thinks there is no proof of affection of the head, and that we have no right to assume that certain individual symptoms, such as the crouping noise or bent thumb, must necessarily be followed by affections of the brain.

One fatal case furnished Mr. North with the following appearances on dissection. The vascular system of the brain presented a highly turgid appearance: a small quantity of blood was effused under the dura mater in several parts: the ventricles contained a small quantity of fluid: the whole cerebral mass was particularly firm: the cerebellum was softer than usual at so short a period after death. The thorax was not examined—a most important omission. The only evidence of disease, detected in the abdomen, was seated in the liver: its whole substance was firm: upon the upper surface were three white spots, about the size of a sixpence, which felt, on passing the scalpel over them, like cartilage: the mesentery and intestines were healthy. After this detail, Mr. N. proceeds to reconcile the circumstances of this case with his previous doctrine, by stating, that the paroxysm which destroyed the child was occasioned by a violent and very passionate fit of crying, induced by some interruption of his amusement; and that death took place from a sudden and overwhelming rush of blood to the head, differing but little from an attack of sanguineous apoplexy in the adult. This child had been in good health for a fortnight before his decease, and his symptoms had more than once vanished by freely lancing the gums. In all cases of this kind, we would urge the necessity of examining the state of the thoracic viscera, after death; and more particularly the nerves distributed to the respiratory system, and the dorsal and cervical portions of the spinal cord.

Chap. VI.—*Treatment of the Spasmodic Affection last described.*—This peculiar affection is regarded by Mr. North as being, in a great majority of cases, connected with a painful and tardy process of dentition; and, in consequence, he recommends a free division of the gums in every case where they are swoln or much expanded upon the surface. He advises the gum being perfectly divided down to the advancing tooth; and, if the incision is made with a lancet, which has "lost its edge," relief may be more confidently expected. A simple and free division of the parts is sufficient for extricating the incisor and canine teeth; but, he says, the molars require a conical incision: he holds it to be worse than useless, to administer anti-spasmodic medicines so long as the local irritation exists. When constipation of the bowels prevails, as it generally does, he employs the more active purgatives: of these, calomel and jalap, or infusion of senna with tincture of jalap are to be preferred. If there is evidence of hepatic derangement and the stools have an unnatural appearance, a grain of calomel may be given each night with advantage. On finding reason to apprehend danger from cerebral disorder, blood must be taken from the jugular vein, or by cupping upon the temples: cold applications to the head, he adds, with the occasional use of purgatives, strict attention to the appearance of the gums, and the greatest care to avoid all external and internal stimuli; are the chief points to be regarded. Sedatives are generally unsuitable: when indispensable, Mr. N. prefers the extracts of hemlock, or henbane: we give an excerpt having reference to this subject: it concludes the Work; and, with it, we shall terminate our analytical exposition of its contents.

"I hope," he says, p. 280, "I shall not be thought to have advocated too strongly, in the course of these pages, the employment of sedatives in infantile diseases. I am perfectly aware of their danger when they are unskilfully employed; but I am also of opinion, that children are not unfrequently bled and purged for the purpose of relieving great irritability, when much advantage might be gained by the judicious application of sedatives, under restrictions to which I have before adverted. In some cases, where the convulsive breathing and violent action of the diaphragm were very great, friction upon the chest with a liniment composed of laudanum, spirits of camphor, and soap liniment, three or four times a day, has certainly proved useful. I have seen blisters applied by other practitioners. I have never known them afford relief, and sometimes they have added considerably to the general irritation and sufferings of the child. During the paroxysms of convulsions, which are so frequent in the course of this affection, the same treatment will be required as that which has been already detailed for the more simple form of convulsive affections. It has been stated that the attack generally commences the moment the little patient wakes from its slumbers; and even after the more severe symptoms have passed off, we shall still find the child rising from its sleep with short and convulsive breathing, and with an appearance of much agitation. These slight remains of the affection may continue for several months; and in more than one instance I have known the attack return with all its original severity, in consequence of the child being suddenly awakened either by accidental noise or the imprudence of the nurse. It is of consequence, then, that every child who has suffered from this malady, should be roused from its sleep with gentleness and caution. The same precaution, indeed, is equally necessary in every form of nervous and convulsive diseases, more particularly those which affect the easily excited constitutions of infants and children."

We need not, in conclusion, pronounce a formal judgment on this volume of Mr. North's: the length of our article, and our sentiments interspersed through it, will bespeak our opinion of its importance: it is plain, concise, and practical; bears testimony to the author's ingenuousness and discrimination; and, altogether, appears to be the result of much patient observation, reading, and reflexion.

